# Differential expression of miRNAs associated with pectoral myopathies in young broilers: insights from a comparative transcriptome analysis

**DOI:** 10.1186/s12864-024-09983-9

**Published:** 2024-01-23

**Authors:** Mariane Spudeit Dal Pizzol, Adriana Mércia Guaratini Ibelli, Maurício Egídio Cantão, Francelly Geralda Campos, Haniel Cedraz de Oliveira, Jane de Oliveira Peixoto, Lana Teixeira Fernandes, Fernando de Castro Tavernari, Marcos Antônio Zanella Morés, Ana Paula Almeida Bastos, Mônica Corrêa Ledur

**Affiliations:** 1 Programa de Pós-Graduação em Zootecnia, Universidade do Estado de Santa Catarina, UDESC-Oeste, Chapecó, Santa Catarina Brazil; 2 Embrapa Suínos e Aves, Concórdia, Santa Catarina Brazil; 3 Programa de Pós-Graduação em Ciências Veterinárias, Universidade Estadual do Centro Oeste, Guarapuava, Paraná Brazil; 4 Departamento de Zootecnia, Programa de Pós- Graduação em Zootecnia, Universidade Federal de Viçosa, Viçosa, Minas Gerais Brazil; 5 Present Address: Embrapa Pecuária Sudeste, São Carlos, São Paulo Brazil

**Keywords:** Epigenetics, Wooden breast, White striping, Small RNAs, Chickens

## Abstract

**Introduction:**

White Striping (WS) and Wooden Breast (WB) pectoral myopathies are relevant disorders for contemporary broiler production worldwide. Several studies aimed to elucidate the genetic components associated with the occurrence of these myopathies. However, epigenetic factors that trigger or differentiate these two conditions are still unclear. The aim of this study was to identify miRNAs differentially expressed (DE) between normal and WS and WB-affected broilers, and to verify the possible role of these miRNAs in metabolic pathways related to the manifestation of these pectoral myopathies in 28-day-old broilers.

**Results:**

Five miRNAs were DE in the WS vs control (gga-miR-375, gga-miR-200b-3p, gga-miR-429-3p, gga-miR-1769-5p, gga-miR-200a-3p), 82 between WB vs control and 62 between WB vs WS. Several known miRNAs were associated with WB, such as gga-miR-155, gga-miR-146b, gga-miR-222, gga-miR-146-5p, gga-miR- 29, gga-miR-21-5p, gga-miR-133a-3p and gga-miR-133b. Most of them had not previously been associated with the development of this myopathy in broilers. We also have predicted 17 new miRNAs expressed in the broilers pectoral muscle. DE miRNA target gene ontology analysis enriched 6 common pathways for WS and WB compared to control: autophagy, insulin signaling, FoxO signaling, endocytosis, and metabolic pathways. The WS vs control contrast had two unique pathways, ERBB signaling and the mTOR signaling, while WB vs control had 14 unique pathways, with ubiquitin-mediated proteolysis and endoplasmic reticulum protein processing being the most significant.

**Conclusions:**

We found miRNAs DE between normal broilers and those affected with breast myopathies at 28 days of age. Our results also provide novel evidence of the miRNAs role on the regulation of WS and in the differentiation of both WS and WB myopathies. Overall, our study provides insights into miRNA-mediated and pathways involved in the occurrence of WS and WB helping to better understand these chicken growth disorders in an early age. These findings can help developing new approaches to reduce these complex issues in poultry production possibly by adjustments in nutrition and management conditions. Moreover, the miRNAs and target genes associated with the initial stages of WS and WB development could be potential biomarkers to be used in selection to reduce the occurrence of these myopathies in broiler production.

**Supplementary Information:**

The online version contains supplementary material available at 10.1186/s12864-024-09983-9.

## Background

Science and technology have led to a significant increase in poultry chain productivity in recent decades [1]. However, these advancements have been related to the onset of some physiological problems in broiler chickens [2–4]. The main pathological changes reported have been abnormalities in the chicken muscle tissues, which develop during the growth phase and progressively worsen during the productive life of the animal [5]. Currently, two main problems affecting broilers are the degenerative disorders caused by White Striping (WS) and Wooden Breast (WB) pectoral myopathies [6, 7].

The main feature of WS myopathy is the presence of white stripes that form parallel to muscle fibers on the breast of affected animals [8, 9]. These stripes are mainly composed by adipose tissue, and histological analysis reveals the presence of overlaid muscle lesions such as myodegeneration, necrosis, lymphocyte and macrophage infiltration, fibrosis, lipidosis, and other degenerative changes [9, 10]. On the other hand, WB myopathy is characterized by regenerative myodegeneration, fibrosis and pectoral muscle hardness [11]. WB also causes several microscopic changes, such as irregular and disarranged fibers, infiltration of inflammatory cells, increased collagen deposition in the tissue, and is often accompanied by WS [11, 12].

Both WS and WB disorders do not represent a risk to the consumer´s health; however, they negatively affect the physicochemical characteristics of the meat [10, 11, 13]. Moreover, fillets affected by myopathies tend to be rejected by consumers [9]. These are some of the reasons why the cuts of the affected animals are undervalued and ultimately designated for by-products in the industry. Meat from affected chickens also represents problems during processing, as their muscle are more exudative, in addition to the large deposition of collagen, which significantly impact the texture of the food. Therefore, product correction is needed through industry interventions [14, 15].

The myopathic pectoral muscle causes damage to the entire poultry chain, both due to their low yield caused by cooking and dripping losses and their devalued cuts [16]. Carcass condemnation rates caused by myopathies are reported to be close to 0.8%, preventing the sale of the whole chicken (which has high commercial value) and resulting in estimated economic losses by approximately BRL 5,90 (US$ 1.20) per kilogram of meat, and daily losses of up to BRL 21,800.00 (US$ 4.300,00) in a slaughterhouse in Brazil [17].

Genetics has been considered an important factor for the development of WS and WB in broilers, with moderate to high heritability for WS (h^2^ = 0.18 ± 0.01 to h^2^ = 0.65 ± 0.08) [18, 19] and low heritability for WB (h^2^ = 0.10) [18]. Differences in the occurrence of myopathies were found among fast-growing commercial lines [20, 21]. Several authors have reported that high-breast-yielding broilers are more affected by myopathies than standard broiler lines [7, 10, 18, 19, 22–25].

Transcriptomic analyses of the pectoralis major muscle (PMM) have provided the identification of the messenger RNA (mRNA) expression profile in broilers affected by myopathies [25–30]. These functional studies have pointed out several candidate genes for the development of these disorders. However, the contribution of epigenetic factors to the development of breast myopathies in chickens are still a challenge, since only one study has associated miRNAs profile with the manifestation of WB myopathy [31] to date.

Given the significance of miRNAs in muscle development and their potential role in the regulation of myopathies in other species [32–34], this study aimed to identify differences in the expression profile of miRNAs between normal broilers and those affected by WS and WB. Additionally, this study seeks to evaluate the potential of miRNAs’ impact on metabolic pathways associated with the onset and differentiation of pectoral myopathies in 28-day-old broiler chickens.

## Results

### Pathological findings

**Fig. 1:**
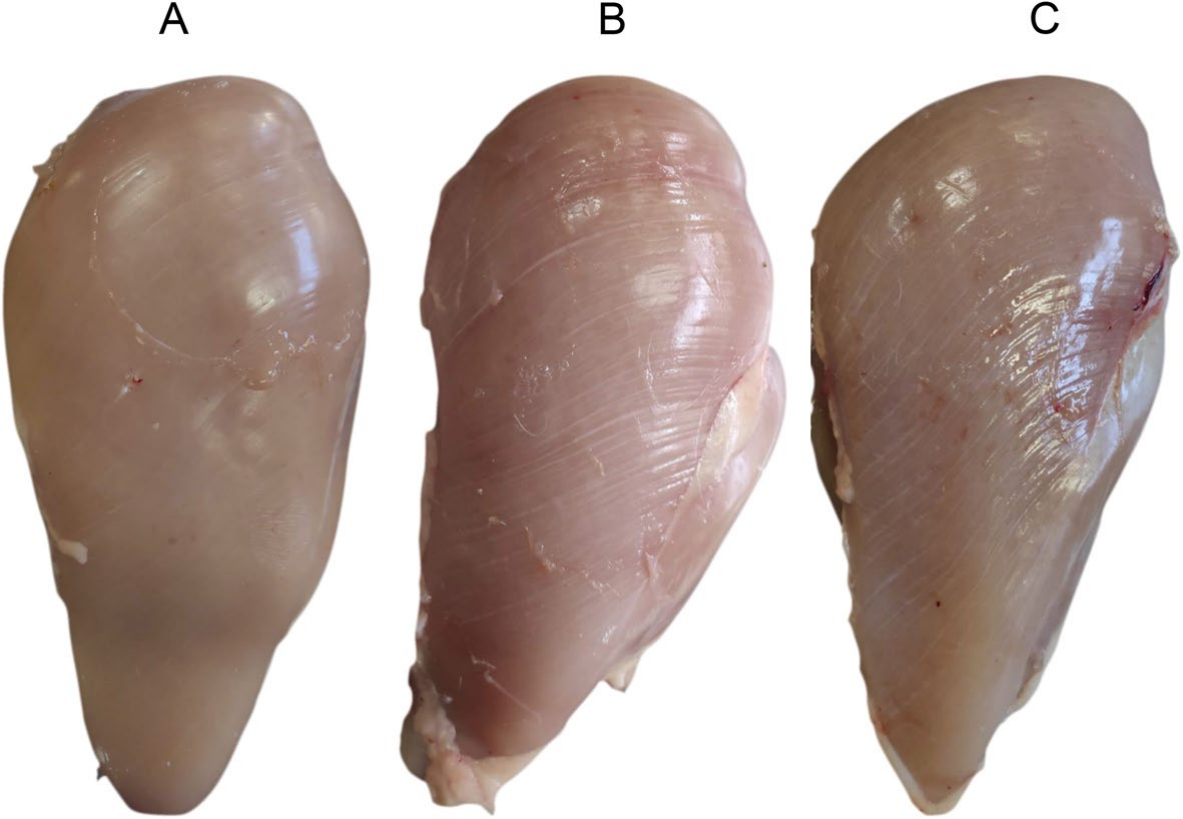
Breasts from 28-day-old broilers representing the macroscopic evaluation of the normal (control) (**A**), white striping (**B**) and wooden breast (**C**) groups

From the 30 pectoralis major muscle evaluated, it was possible to classify 27 of them: 4 as normal (no apparent macroscopic lesions), 16 with WS and 7 with WB, according to the classification criteria established by Kuttappan et al. (2013) and Sihvo, Immonen and Puolanne (2014) (Fig. 1).

**Fig. 2:**
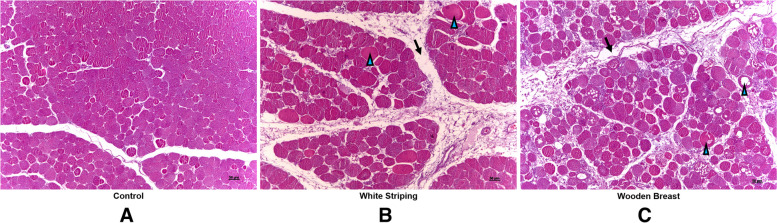
Histopathological analysis of 28-day-old chicken breasts showing microscopic features of the control (**A**), white striping (**B**), and wooden breast (**C**) groups. Increase in space between muscle bundles (arrow), several degrees of degenerated fibers (arrowhead)—Haematoxylin and eosin stain

The histopathological evaluation of 27 out of 30 initial samples revealed 4 normal muscle samples showing organized muscle fibers of regular size with rare hypereosinophilic fibers (Fig. 2A). Sixteen (16) samples showed lesions consistent with WS: mild to moderate presence of hypereosinophilic fibers, moderate number of degenerated and necrotic fibers, an increase in the spaces between fibers and muscle bundles and moderate proliferation of intramuscular adipocytes (Fig. 2B). Finally, 7 samples presented WB compatible lesions: high number of hypereosinophilic and necrotic muscle fibers, moderate to high proliferation of fibroblasts, muscle fibers showing different sizes, looser cells arrangement with significant increase in the spaces between fibers and muscle bundles, presence of interstitial connective tissue, mild heterophile infiltration and moderate intramuscular adipose tissue (Fig. 2C).

Based on the macroscopic and microscopic analyses, the muscle samples were classified into three groups: control (none or slight lesions), WS-affected, and WB-affected groups. For miRNA analysis, the most representative samples of each group were selected: three samples for the control group, five samples for the WS-affected group, and six samples for the WB-affected group.

### Sequencing, quality control and mapping

Approximately 133 million reads were sequenced across all samples, resulting in an average of 9.55 million reads per sample. After quality control analysis, a mean of 7.5 million reads per sample remained, which were aligned against the ribosomal (rRNA) and transporter RNAs (tRNA) using RFAM database release 14. Around 1.1% of those sequences were removed for downstream analysis. Then, an average of 67.5% of the sequences were mapped in the *Gallus gallus* genome (GRCg6a, accession GCF_000002315.5, Supplementary File 1: Table S1).

### miRNA identification and differential expression analysis

**Fig. 3:**
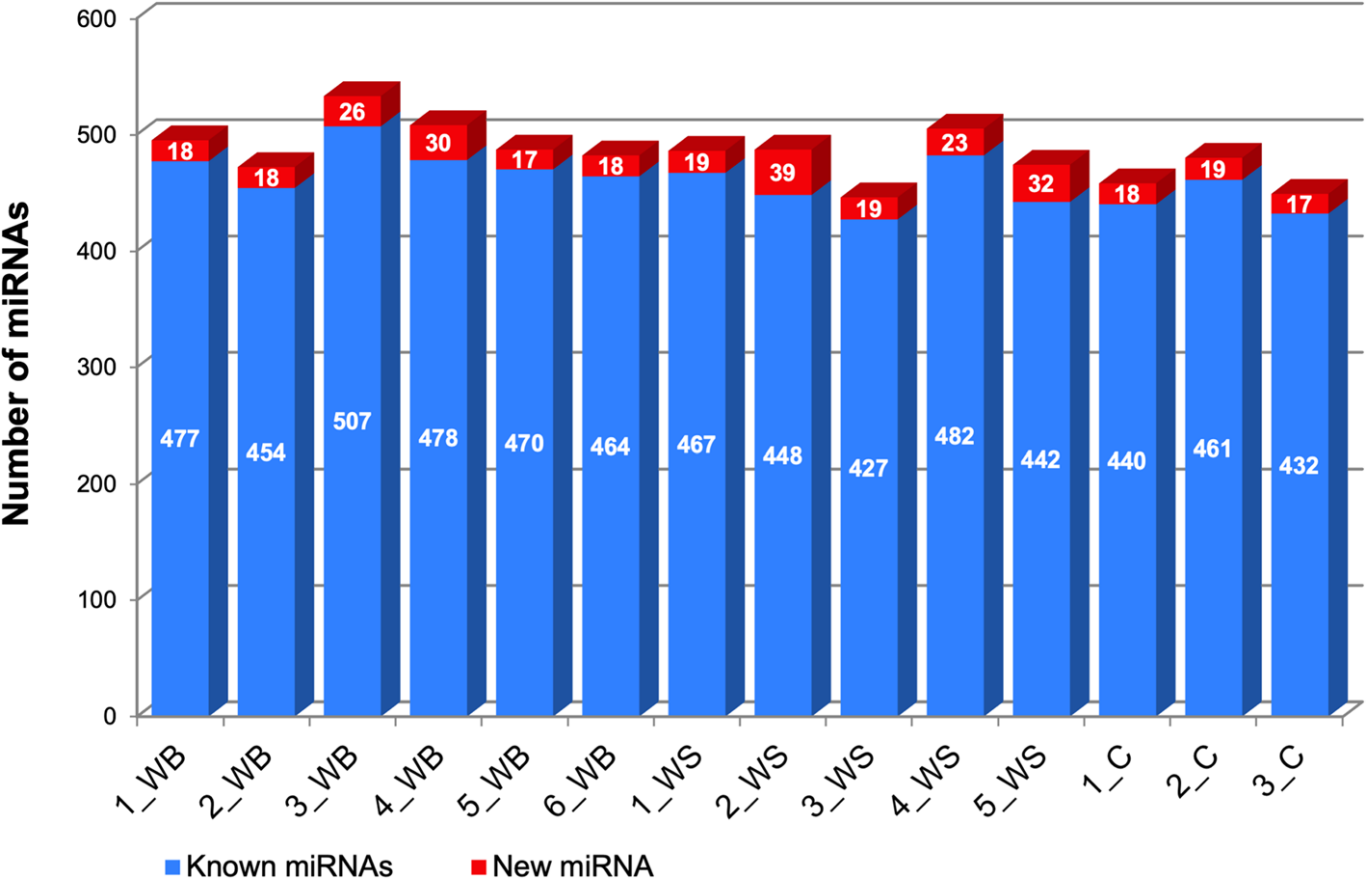
Number of identified (known and new) miRNAs in each sample of pectoralis muscle tissue

A total of 844 miRNAs were detected based on all miRNAs identified by miRDeep2. From those, 755 were known miRNAs and 89 were firstly described in this study (Fig. 3). After filtering the reads with low expression according to the standard "filterbyexpr" function from EdgeR [35], 303 miRNAs were determined as expressed, including 286 known miRNAs and 17 new ones.

**Fig. 4:**
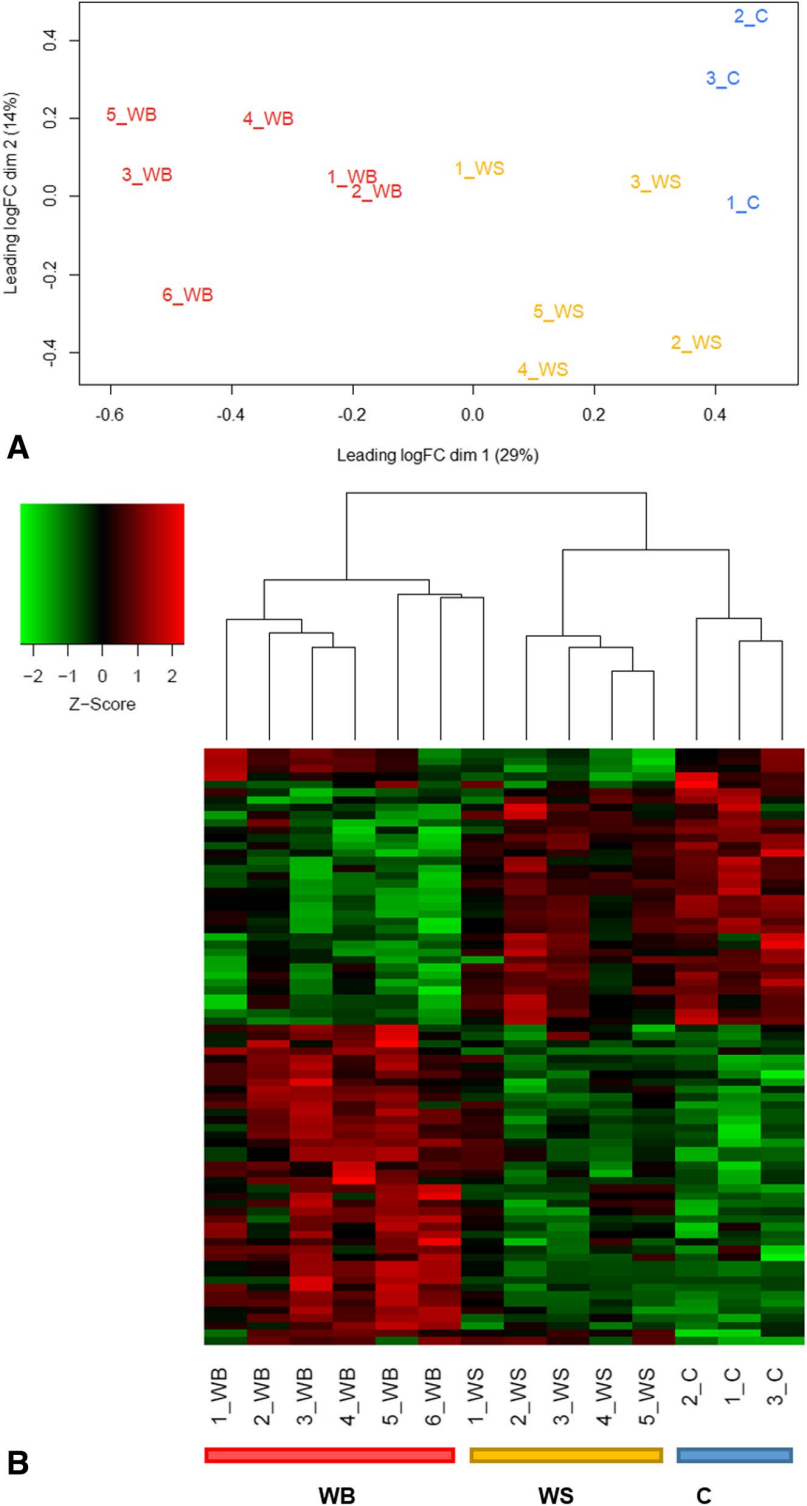
Multidimensional scale (MDS) plot (**A**) and heatmap (**B**) showing the separation of control, white striping (WS) and wooden breast (WB) groups through the miRNA’s expression profile. Heatmap hierarchically grouping the expression of 80 DE miRNAs that most differed among the 3 groups. The intensity of the color represents the degree of regulation (upregulated in red and downregulated in green)

A multidimensional scaling plot (MDS) was generated based on the profile of expressed miRNAs, and the three groups were separated according to their respective physiological conditions (Fig. 4A). This result shows a consistent miRNA profile in the samples within each group, indicating homogeneity. Similar separation pattern was also observed in the heatmap (Fig. 4B).

For the DE analysis, three comparisons were performed: WS vs control group, WB vs control group and WB vs WS group. Considering WS vs control, five miRNAs were DE, four downregulated and one upregulated in the WS group (Table 1).

**Table 1:** Differentially expressed miRNAs between 28-day-old control and white striping-affected broilers

miRNAs	logFC	logCPM	*p*-value	FDR
gga-miR-375	-4.48	3.51	7.22E-06	0.0022
gga-miR-200b-3p	-2.90	4.84	0.00021	0.0230
gga-miR-429-3p	-2.56	4.25	0.00023	0.0230
gga-miR-1769-5p	3.67	0.37	0.00057	0.0435
gga-miR-200a-3p	-2.74	7.17	0.00074	0.0451

*logFC* log fold-change, *logCPM* log copy per million, *FDR* False discovery rate

When comparing WB and control groups, 82 miRNAs were DE; 43 upregulated and 39 downregulated in the WB-affected group (Table 2, Supplementary file 1: Table S2).

**Table 2:** Top 5 up and downregulated miRNAs in the WB-affected compared to the control group

	**miRNA**	**logFC**	**logCPM**	***P*Value**	**FDR**
Upregulated	chr22_10817	5.51	-0.29	0.0014	0.0089
	gga-miR-1769-5p	3.69	0.36	0.0003	0.0033
	gga-miR-3530-3p	3.68	0.69	0.0001	0.0013
	gga-miR-205a	3.30	3.51	0.0009	0.0064
	gga-miR-222b-5p	2.60	1.34	8.26E-07	4.17E-05
Downregulated	gga-miR-6553-5p	-1.79	1.21	0.0058	0.0274
	gga-miR-6553-3p	-1.87	3.97	0.0013	0.0086
	chr2_9820	-2.00	3.83	2.79E-07	2.11E-05
	chr2_9097	-2.72	0.78	0.0004	0.0033
	gga-miR-122-5p	-4.42	9.15	0.0066	0.0294

Considering the comparisons of the two affected groups WB with WS, 61 miRNAs were DE, 37 upregulated and 24 downregulated in the WB group (Table 3, Supplementary file 1: Table S3).

**Table 3:** Top 5 up and downregulated miRNAs in the WB compared to WS-affected group

	**miRNA**^a^	**logFC**	**logCPM**	***P*Value**	**FDR**
Upregulated	chr22_10817	5,51	-0,29	3,45E-05	0,000826
	gga-miR-1663-5p	4,72	0,14	3,89E-09	1,18E-06
	gga-miR-200b-3p	3,25	4,83	7,68E-06	0,000258
	gga-miR-375	3,21	3,50	0,000298	0,004297
	gga-miR-200a-3p	3,12	7,17	3,55E-05	0,000826
Downregulated	gga-miR-144-3p	-1,20	5,34	0,0016	0,016015
	gga-miR-193a-3p	-1,24	3,65	0,0004	0,0061
	gga-miR-451	-1,32	9,25	2,41E-05	0,000724
	chr2_9820	-1,41	3,83	5,48E-05	0,001186
	chr2_9097	-1,89	0,78	0,007947	0,043001

^a^miRNA names starting with “chr” are predicted for the first time in this study

**Fig. 5:**
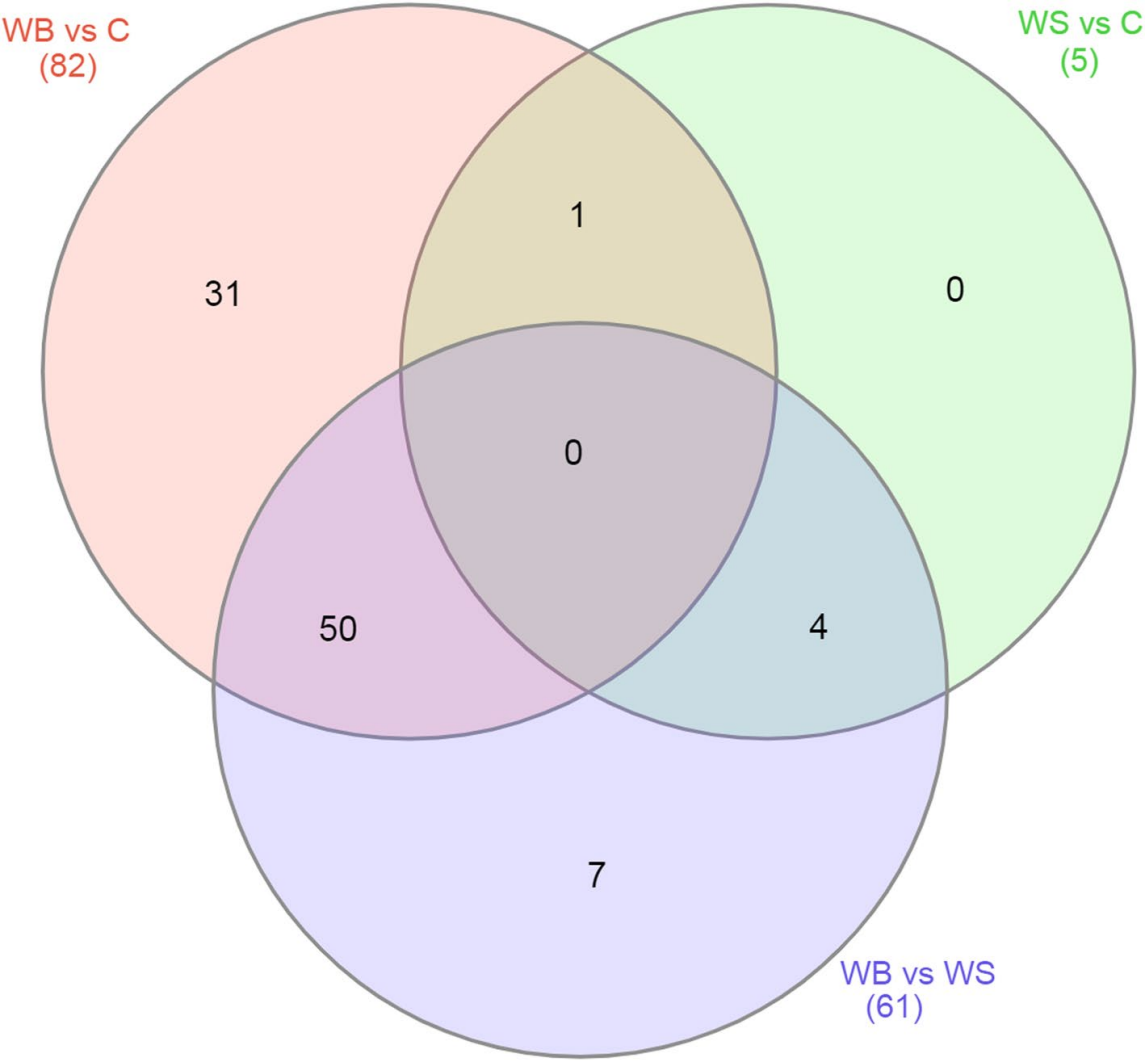
Venn diagram showing the number of miRNAs differentially expressed in comparisons between each contrast

Evaluating the three contrasts (Fig. 5), the Venn diagram showed that 31 miRNAs were exclusively DE between WB-affected and the control group, 7 miRNAs were DE only between WB and WS, and no miRNA was exclusively DE in the WS vs control group comparison.

### Functional annotation

Once the DE miRNAs were identified, the sRNAtoolbox and ShinyGO tools were used to predict the target genes for these miRNAs in each of the following contrasts:

#### White striping-affected versus control group

Evaluating the five miRNAs DE in this comparison (Table 1), 2176 target genes were found in the chicken genome, out of which 2131 were previously annotated and used for gene ontology analysis. Eight pathways were enriched with the target genes predicted for WS (Table 4, Supplementary File 1: Table S4), with autophagy and endocytosis as the most significant pathways.

**Table 4:** Metabolic pathways regulated by target genes of differentially expressed miRNAs in 28-day-old broilers affected by White Striping compared to the control group

Pathways	Identified genes	Pathway Genes	FDR
*ERBB* signaling pathway	20	76	1.0E-02
Autophagy	33	126	7.8E-04
Insulin signaling pathway	29	114	1.7E-03
*FoxO* signaling pathway	28	119	7.6E-03
Cell cycle	25	114	3.3E-02
Endocytosis	49	225	7.8E-04
*MTOR* signaling pathway	28	137	4.2E-02
Metabolic pathways	185	1304	4.2E-02

#### Wooden breast-affected versus control group

Out of 82 DE miRNAs obtained from this contrast, 7148 target genes were found in the chicken genome, which enriched 20 metabolic pathways, such as Ubiquitin mediated proteolysis, Protein processing in endoplasmic reticulum, Cell cycle, Endocytosis, Autophagy, Insulin signaling pathway and FoxO signaling pathway (Table 5, (Supplementary File 1: Table S5).

**Table 5:** Metabolic pathways regulated by target genes of miRNAs differentially expressed in Wooden Breast compared to the control group

Pathway	Identified genes	Pathway Genes	FDR
Fatty acid degradation	23	32	1.3E-03
Fatty acid metabolism	35	51	2.5E-04
Biosynthesis of nucleotide sugars	24	36	5.0E-03
Amino sugar and nucleotide sugar metabolism	29	45	3.6E-03
Cysteine and methionine metabolism	27	42	5.0E-03
Peroxisome	50	79	1.9E-04
Ubiquitin mediated proteolysis	80	129	2.7E-06
Protein processing in endoplasmic reticulum	89	149	4.5E-06
Cell cycle	67	114	2.0E-04
Endocytosis	131	225	2.4E-07
Autophagy	73	126	1.9E-04
Cellular senescence	78	137	3.4E-04
Biosynthesis of cofactors	68	120	5.8E-04
Lysosome	64	114	1.2E-03
Insulin signaling pathway	64	114	1.2E-03
FoxO signaling pathway	66	119	1.3E-03
Carbon metabolism	53	96	5.0E-03
Tight junction	78	145	1.3E-03
Salmonella infection	117	226	5.0E-04
Metabolic pathways	647	1304	7.8E-15

## Discussion

The regulatory role of miRNAs in myopathies has been previously reported in humans and other species [36, 37], but very limited research exists for chickens [31]. Our study focused on 28-day-old broilers, aiming to investigate early stages of WS and WB. These findings shed light on mechanisms linked to milder conditions and potential onset, underscoring the prevalence of these issues in fast-growing commercial chicken lines, even before slaughter age. It is important to emphasize that among the 30 samples, only four were classified as controls due to the challenge of finding birds without macroscopic and histological myopathy lesions.

In the differential expression analysis, five miRNAs were DE between WS-affected and control groups, while 82 were DE between WB-affected and control group. Some of those 82, such as gga-miR-146-5p, gga-miR-29, gga-miR-21-5p, gga-miR-133a-3p and gga-miR-133b have already been associated with WB in broilers at 42 days of age [31]. However, our study marks the first association of miRNAs with the regulation of WS myopathy in broilers. Four out of five DE miRNAs in the WS-affected broilers vs control group (gga-miR-375, gga-miR-200a-3p, gga-miR-200b-3p and gga-miR-429-3p) were also DE when Chao et al. [38] evaluated high-fat and low-fat chickens. It has been shown that the upregulation of miR-429-3p was correlated with *LPIN1* downregulation, promoting abdominal fat accumulation through the PPARγ pathway [38]. Hence, we have found miRNAs associated with the regulation of adipogenesis, a key biological process for the development of the WS phenotype.

Once the DE miRNAs were identified, the target genes were predicted, and metabolic pathways were functionally analyzed. The discussion initially focused on common pathways between WB and WS to reveal shared mechanisms. Subsequently, the most important exclusive pathways for each myopathy were explored.

### Shared metabolic pathways enriched in WB and WS-affected groups:

Among the six common pathways, three were selected for discussion: autophagy, insulin signaling, and *FoxO* signaling.

#### Autopaghy

This was one of the most significant metabolic pathways involved in both conditions compared to the control group (Tables 4 and 5). Autophagy is a cellular process that involves the degradation and recycling of cellular components, promoting cell survival and maintaining homeostasis. It plays a crucial role in eliminating damaged organelles and proteins and responds to cellular stress [39–45]. Dysregulation of this mechanism can cause tissue degradation leading to degenerative diseases, when upregulated [46], or the accumulation of harmful substances, fostering the replication of defective cells, when downregulated [46].

A total of 73 miRNAs’ target genes were enriched in this BP in the WB vs control comparison, and 33 in the WS vs control contrast. Notably, gga-miR-155, gga-miR-146b, and gga-miR-222, previously identified as upregulated in 42-day-old broilers affected with WB [31], were prominent and also over-expressed in the WB vs control contrast. These miRNAs, when highly expressed, may have implications in numerous human muscle disorders [47]. miR-155 is a multifunctional miRNA that modulates autophagy through decreasing the expression of Autophagy related 5 gene (*ATG5*) [48, 49]. This gene, in association with the autophagy related 12 gene (*ATG12*), another predicted target, contributes to the structural maintenance and maturation of autophagosomes [50–52]. An indirect evidence of an ongoing impairment of the autophagic process is the identification of miRNAs targeting genes involved with myoblast differentiation: miR-155, which targets myocyte-specific enhancer factor 2A (*MEF2A*) [34] and miR-146 targeting the Mothers Against Decapentaplegic Homolog 4 (*SMAD4*), Neurogenic Locus Notch Homolog Protein 1 (*NOTCH1*) and High Mobility Group Protein HMGI-C (*HMGA2*) genes [34]. It has been shown that hindering the myocyte fusion in the final stages of myoblast differentiation leads to an impairment in the autophagosomes biogenesis [53]. Moreover, miRNAs 222b-3p and gga-miR-222b-5p play roles in apoptosis regulation [39], a BP that has already been associated with WS development [29]. In this sense, FADD Like Apoptosis Regulator (*CFLAR*), an exclusive WB target, coordinates autophagy, apoptosis, and necroptosis [40]. Since the *CFLAR* mRNA could be degraded by the activity of miRNAs, an increase in tissue autophagy is expected.

Autophagic dysregulation in WS and WB leads to degenerative lesions, indicating muscle damage with an endogenous origin [11, 41]. This dysregulation could predispose broilers to myopathies. Both WS and WB exhibit fibrotic tissue and degenerative lesions [9, 10], highlighting the critical role of autophagy regulation in the development of these myopathies.

#### Insulin signaling pathway

Chicken affected by WB and WS exhibit elevated lipid content in the pectoral muscle [9, 54, 55], suggesting a potential association between increased fat deposition and the regulation of the insulin pathway. Ebrahimi et al. [42] demonstrated that post-transcriptional mechanisms regulate the insulin pathway, contributing to disorders like insulin resistance and obesity in humans. In the WB vs control comparison, two members of the suppressor of cytokine signaling (SOCS) family, *SOCS3* and *SOCS4* were enriched in this pathway. *SOCS3* acts on inflammatory processes, whereas *SOCS4* is involved in the regulation of hormones like insulin and growth factors [56]. Studies have shown that increased expression of miR-203 reduces *SOCS3* levels in humans, evincing the translational control over SOCS gene members by miRNAs [57]. miR-203, upregulated in the WB broilers, is a known regulator of insulin sensitivity, glucose tolerance, and subcutaneous white adipose tissue accumulation [58, 59]. Its upregulation might be linked to the impairment of the glucolipotoxicity pathway, previously associated with the etiology of WB and other breast myopathies in broilers [59].

An exclusive target for WB was the CBL Proto-Oncogene B (*CBLB*) gene, which acts in the proteasome-mediated protein degradation [60], and is regulated by miR-29 [51]. In our study, several miRNAs of this family were upregulated in the WB-affected group, including gga-miR-29a-5p, gga-miR-29a-3p, gga-miR-29c-3p, gga-miR-29b-1-5p and gga-miR-29b-3p. They were previously found to be DE in WB-affected broilers from a commercial line at 42 days of age, and it is believed that the gga-miR-29 has a role in the WB development through energy metabolism regulation [31].

Insulin resistance is a key factor in metabolic disorders [42], and it has been shown that miRNAs can regulate the expression of the insulin pathway and insulin resistance [42, 61]. Problems in insulin signaling in the liver have been linked to lipidosis [42], as emphasized by Lake and Abasht [59]. Therefore, our study suggests that miRNAs may regulate genes in the insulin pathway, potentially contributing to the development of WS and WB in broilers. The miR-15b has already been directly associated with insulin resistance [52] and here, we found that gga-miR-15b-3p was among the DE miRNAs between WB and control group. Furthermore, gga-miR-222b-3p and gga-miR-222b-5p were upregulated in the WB group, and its upregulation has already been associated with induced insulin resistance in mice [62]. These findings indicate that these mechanisms possibly alter the insulin pathway also in broiler chickens, facilitating the myopathies occurrence.

#### FoxO signaling pathway

A total of 28 and 66 target genes from the WS vs control and WB vs control comparisons, respectively, were enriched in the FoxO signaling pathway using the ShinyGO tool. Among the regulators of this pathway is miR-146b, which suppresses *FoxO1* and *FoxO3* genes, promoting adipogenesis in tissues [63]. In the current study, gga-miR-146b-5p and gga-miR-146b-3p were upregulated in the WB-affected group, potentially contributing to increased body weight and adipose tissue. Conversely, miR-130 suppresses adipogenesis [64] and, in our study, gga-miR-130a-3p was downregulated in WB-affected broilers, which could favor lipid deposition.

*FoxO1* and *FoxO3* genes are also related with vascular development [65, 66] and their absence can lead to severe cardiovascular anomalies in animals. Vascular tissue impairment has already been associated with myopathic conditions [67]. FoxO signaling is also activated in response to stress and *FoxO3* is associated with the induction of autophagy [68]. Studies have shown that miR-132 regulates *FoxO3* expression, acting as anti-hypertrophic and pro-autophagic [69]. Notably, gga-miR-132a-5p, gga-miR-132c-5p and gga-miR-132c-3p were upregulated in the WB-affected group. Additionally, miR-30d, targeting FoxO3 and associated with reduced inflammatory cell death [70], was downregulated in the WB-affected group, along with other family members like gga-miR-30a-3p, gga-miR-30e-5p, gga-miR-30a-5p, gga-miR-30c-5p, and gga-miR-30c-1-3p.

### Exclusively enriched pathways in the WS-affected compared with the control group

#### ERBB signaling pathway

The ERBB family, among other functions, guides cell–cell interactions in tissues and organ formation during animal growth [71]. Most cells have more than one type of ERBB receptors [72]. In the WS-affected group, approximately 20 target genes in this pathway, including MAPK family members (*MAPK10*, *MAP2K4*, and *MAPK9*), were identified through DE miRNAs. Notably, miR-375, downregulated in the WS-affected group, and its target genes *ERBB2* and *MAPK* were involved in fat metabolism and considered as adipocyte markers [73]. Dysregulated ERBB signaling, reported in kidney disease [74], contributes to epithelial hyperproliferation, inflammation, and fibrosis, which is a hallmark of WS.

The ERBB signaling pathway might be connected with changes in WS through the identification of DE miRNAs known for suppressing the expression of ERBB receptors [75]. mir-375 plays a role in initiating apoptosis via ERBB2 receptor expression, and its downregulation triggers cell proliferation and tumorigenesis [75]. The downregulation of this miRNA could favour cell proliferation in the WS-affected broilers. Furthermore, abnormal expression of ERBB pathway were also related with inflammation and fibrosis appearance, two features observed in chickens affected with WS [10, 76].

#### mTOR signaling pathway

The mTOR signaling pathway is key in BP related to cell growth, survival, aging and healthy muscle development [77, 78]. The mTOR positive regulation is related with muscular hypertrophy [78, 79]. The mTOR regulates insulin sensitivity [80] and integrates information from the extracellular environment, such as availability of nutrients and energy, into intracellular stimuli promoting protein synthesis [81]. Twenty-eight target genes from DE miRNAs between WS and control group enriched the mTOR signaling pathway. The miR-375 plays an important role in the mTOR pathway suppressing cell proliferation and apoptosis [82], and also inhibiting cellular signals of osteogenesis and adipogenesis [4]. This miRNA, also known to control adipogenesis and regulate mTOR-mediated autophagy [83], was upregulated in the control group, potentially limiting adipogenesis in normal broilers and allowing greater lipid deposition in WS-affected broilers muscles. The downregulation of miR-375 and two miR-200 family members in WS-affected broilers may contribute to increased adipogenesis in pectoral muscles [83].

Another downregulated miRNA in the WS-affected broilers was the gga-miR-429-3p. This miRNA family is known to be downregulated during hypoxia [84], suggesting a potential association with increased hypoxia levels in chickens with WS [76]. Among all the functions that have been identified for the mTOR pathway, it also regulates glucose resistance, cell proliferation and autophagy. Our results support the hypothesis that WS may result from disruptions in glucose and lipid metabolism, aligning with the hypothesis proposed by Lake and Abasht [59].

### Exclusively enriched pathways in the WB-affected broilers compared to the control group

#### Ubiquitin-mediated proteolysis (UP)

The Ubiquitin–Proteasome (UP) system degrades intracellular proteins and structures dispersed in the cytosol with high specificity [85], and 80 genes were predicted to be targets of regulation by the miRNAs DE between WB and control groups. MiR-122, identified in hypoxic skeletal muscles, participates in the Ubiquitin-mediated proteolysis pathway, and contributes to the development of musculoskeletal diseases, such as myofibrillar degradation [86]. In WB-affected broilers, two downregulated members of the miR-122 family (gga-miR-122-5p and gga-miR-122b-5p) were identified and potentially linked to histological lesions observed in the pectoral muscle, such as increased necrosis levels and myofiber degeneration.

Abnormal UP pathway activity can induce pathological conditions like muscle atrophy [11, 87–90] and accumulation of oxidized proteins [88]. Additionally, it can trigger several anomalies in skeletal muscle, including basophilic infiltrations, degenerative and regenerative alterations [89]. Most of these microscopic features are observed in WB myopathy [11]. Therefore, it is reasonable to assume that miRNAs likely influence the regulation of the proteolysis pathway in WB-affected muscle, given the UP system’s high activity during myogenesis and its role in muscle development [90].

#### Protein processing in the endoplasmic reticulum

The endoplasmic reticulum (ER) plays a crucial role in producing integral and secretory proteins for the plasma membrane [91]. Eighty-nine genes involved in ER protein processing pathway were identified as targets of DE miRNAs between WB and the control group. Studies indicate that certain miRNAs inhibit mRNAs translation in the ER, directly interfering protein synthesis and processing, thus influencing organismal development [92]. miRNAs form a complex regulatory network in this pathway, for instance, miR-122 can act in UP and apoptosis [39]. In our study, gga-miR-122-5p was downregulated in broilers with WB, suggesting its potential influence on WB manifestation.

Some miRNAs, including miR-29 [93], respond to ER stress conditions by regulating pro-apoptotic genes and influencing cell death [39]. In our study, we found six mirRNAs of this family overexpressed in broilers with WB compared to the control group (gga-miR-29a-5p, gga-miR-29a-3p, gga-miR-29c-3p, gga-miR-29b-1-5p and gga-miR-29b-3p). Furthermore, the gga-miR-455-5p, a miRNA linked to transcription factors involved in ER homeostasis [39], was also upregulated in broilers with WB. Although not previously associated with myopathic disorders, this miRNA might affect ER homeostasis in WB-affected chickens. ER stress, associated with degenerative disorders and myopathies, may originate from glucose and nutrient deprivation, hypoxia, inflammation and oxidative stress. High ER stress levels have already been related to the development of myopathies [94, 95]. Moreover, myopathic features, such as cell death, regenerative changes and muscle weakness were also related to ER stress [96].

Several changes that cause ER stress are observed in chickens with WB, such as hypoxia and oxidative stress [11, 54]. These conditions serve as sources of ER stress in the pectoralis muscle, disrupting protein synthesis and processing in the ER. These observations strongly suggest an important role of the ER protein processing pathway in the manifestation of WB.

## Conclusions

Our study identified hallmark lesions in both WS and WB myopathies. The miRNA expression profile unveiled only one shared DE miRNA in both conditions compared to the control, suggesting that the molecular mechanisms underlying these two myopathies may differ, given the limited overlap in DE miRNA. Notably, our results provide a novel evidence of the involvement of miRNAs in regulating WS and in the differentiation of both WS and WB myopathies. Additionally, when comparing WB and WS-affected vs the control group contrasts, WB-affected broilers exhibited a higher number of DE miRNAs, suggesting a stronger influence of miRNA control in broilers affected with WB than with WS. These findings underscore the role of epigenetic factors in regulating both myopathies. Furthermore, functional enrichment and ontology analysis of DE miRNA target genes implicated specific metabolic pathways in the manifestation of these myopathies. Our results highlight the miRNAs’ role in energy and insulin metabolism, hypoxia, autophagy, inflammation, protein synthesis and cell proliferation mechanisms. Overall, our study provides valuable insights into the miRNAs and pathways associated with the occurrence of WS and WB myopathies at an early age, which can possibly help developing new approaches to reduce these myopathies by adjustments in nutrition and management. Furthermore, the identified miRNAs and target genes are potential biomarkers to be used in selection to reduce these conditions in broiler production.

## Methods

### Animals and sample collection

This work was carried out at the Embrapa Swine and Poultry National Research Center, located in Concórdia—Santa Catarina State, Brazil. Thirty Ross male broilers were reared in boxes and managed according to the commercial line recommendations, receiving standard feed and water ad libitum. The broilers were euthanized by cervical dislocation at 28 days of age, following the practices recommended by the Committee on Ethics in the Use of Animals (CEUA protocol 08/2019). Immediately after slaughter, the *pectoralis major muscle* (PMM) of the chickens were visually evaluated for the presence or absence of WS and WB, according to KUTTAPPAN et al. (2013) [76] and SIHVO; IMMONEN; PUOLANNE (2014) [11]. Approximately 1 cm^2^ of the PMM was collected from the cranial region for histopathological and miRNA sequencing analyses.

### Histopathological analyses

For the histopathological analyses, the collected samples were fixed in 4% paraformaldehyde until processing. Tissues were cut into 5 mm sections, dehydrated in alcohol, diaphanized and embedded in paraffin. Then, tissues were cut into 3 μm sections, mounted in slides and stained with hematoxylin and eosin for morphologic evaluation and identification of myopathic lesions.

### RNA extraction, library preparation and sequencing

RNA extraction was performed from 100 mg of pectoral muscle samples, which were ground with a mortar and pestle in liquid nitrogen. Then, the total RNA was extracted using the Trizol protocol, according to the manufacturer's instructions. Total RNA was quantified in a BioDrop spectrophotometer (Biodrop, UK), and was considered of good quality when the OD260: OD280 ratio was greater than 1.8. The integrity of the samples was confirmed by electrophoresis for 90 min in a 1% agarose gel and also using a Bioanalyzer Agilent 2100 equipment, where samples with RNA Integrity Number (RIN) greater than 8 were used for downstream analyses.

The miRNA libraries were constructed using QIAseq miRNA Library kit (Qiagen, Germany) with the standard protocol. Libraries were quantified and verified in the Bioanalyzer Agilent 2100 equipment and by quantitative PCR (qPCR). Sequencing was carried out in NextSeq 2000 equipment (Illumina), at the Life Sciences Core Facility (LaCTAD) of the University of Campinas (UNICAMP), in Campinas, São Paulo State, following a single-end protocol (1 × 75 bp).

### Sequencing quality control and mapping

The FASTQ files were submitted to quality control (QC) analysis using the Trimmomatic tool [97] in order to remove sequences with low average Phred quality score (PHRED < 20), short reads (length < 18 nucleotides) and sequences with undefined bases (identified as N). Following, the unique molecular identifiers (UMIs) were extracted and deduplicated using the UMI-tools [98]. Then, an initial mapping using bowtie [99] was performed against the Rfam database release 14 (https://rfam.org/) [100] to remove tRNA and rRNAs sequences. After that, the miRDeep2 software [101] was used to map the remaining sequences against the chicken genome (GRCg6a, accession GCF_000002315.5) to identify and quantify miRNA sequences present in the analyzed samples. Furthermore, the miRDeep2 was also applied to discover potential novel chicken miRNAs. For quantification of known miRNAs, FASTA files from miRBase release 22.1 [102] and MirGeneDB release 2.1 [103, 104] databases were used. These analyses were run in the BAQCOM automated pipeline (https://github.com/hanielcedraz/BAQCOM).

### Reads counting, filtering, miRNA differential expression and functional annotation

The miRNA counts were obtained using the miRDeep2 software [101] and the counts were filtered using the "filterbyexpr" function from the edgeR package [35] from R language (R Core Team, 2015). Then, the remaining miRNAs were also analyzed with edgeR for differential expression among the three groups (control, WS and WB). miRNAs with false discovery rate (FDR) < 0.05 were considered DE, after correcting for the Benjamini-Hochberg (BH) multiple-test. After obtaining DE miRNAs, the target mRNAs were searched using the sRNAtoolbox [105] online tool, with the default parameters for the Pita, miRanda, TargetSpy and Simple Seed Analysis tools. The miRNAs target genes were submitted to gene ontology analysis with the ShinyGO software [106].

### Supplementary Information

**Additional file 1.**

## Data Availability

The datasets analyzed in this study are available from the corresponding author on reasonable request. The miRNA sequences are available in the SRA database under the BioProject number PRJNA950417 (https://dataview.ncbi.nlm.nih.gov/object/PRJNA950417?reviewer=43oo1m5jtro2jsi4du00bv8r8r; these files will be released upon publication).
